# Specific detection of three *Torradovirus *species with digoxigen-labeled probes

**DOI:** 10.1186/1753-6561-8-S4-P125

**Published:** 2014-10-01

**Authors:** Dorivaldo Marques da Silva Junior, Inmaculada Ferriol, Bryce Falk

**Affiliations:** 1 CAPES Foundation, Ministry of Education of Brazil, Brasília - DF, Zip Code 70.040-020 - Brazil; 2 Department of Plant Pathology, University of Davis California, 95616, CA, USA

## Background

Torradoviruses are an emerging group of picorna-like plant virus from the family Secoviridae that infect tomato and other Solanaceae species. The genus *Torradovirus *include four species: *Tomato torrado virus *(ToTV), first found in Europe, and afterward in Central America and Australia; *Tomato apex necrosis virus *(ToANV), present in Mexico; *Tomato chocolate spot virus *(ToChSV) and *Tomato chocolate virus *(ToChV), both found in Guatemala. The symptoms caused by these viruses include chlorotic regions on the leaves that may develop to necrotic spots and holes, while the fruits show necrotic lines and frequently cracks on the surface, reducing yield and quality [1]. Even though these viruses have not yet been found in Brazil, the sudden spread of ToTV from Europe to Australia and Central America emphasize the necessity of effective methods to detect them. For that reason, we have developed digoxigenin-labelled RNA probes for the detection of three torradoviruses, ToANV, ToChSV and ToTV, through hybridization.

## Methods

In order to produce those riboprobes, total RNA was extracted from infected *Nicotiana Benthamiana *plants using TRIzol Reagent and used for RT-PCR with the generic primers pair described by Verbeek and colleagues [2], modified with the addition of the T7 promoter to the 5' end of the reverse primers. The RT-PCR products were purified and transcribed *in-vitro *using MEGAscriptT7kit (Invitrogen) and digoxigenin-labeled nucleotides. The RNA probes produced were purified and hybridized to total RNA extracted from plants infected with which one of the viruses and non-infected plants blotted onto nylon membranes. Anti-digoxigenin Fab fragments conjugated to alkaline phosphatase were bound to the hybridized digoxigenin-labeled probes and the chemiluminescent substrate CDP-Star was added in order to produce light. X-ray films were exposed to the membranes and developed.

## Results and conclusions

The X-ray films showed that each probe was able to hybridize only to the target virus, while no hybridization was observed for the RNA extractions from non-infected plants or plants infected with other viruses. In conclusion, these results show that the digoxigenin-labeled RNA probes can be used for the effective and accurate detection of the three *Torradovirus *species.

## References

[B1] Gómez P Sempere RN Amari K Gómez-Aix C Aranda MA Epidemics of Tomato torrado virus, Pepino mosaic virus and Tomato chlorosis virus in tomato crops: do mixed infections contribute to torrado disease epidemiology? Ann Appl Biol 2010 156 401 410 10.1111/j.1744-7348.2010.00397.x

[B2] Verbeek M Tang J Ward LI Two generic PCR primer sets for the detection of members of the genus Torradovirus J Virol Methods 2012 185 184 188 10.1016/j.jviromet.2012.06.020 22771385

